# Quantitative mid-treatment imaging biomarkers for response prediction after radiotherapy in head and neck cancer: a systematic review and meta-analysis

**DOI:** 10.1186/s13550-026-01446-4

**Published:** 2026-06-06

**Authors:** Yuvnik Trada, Mark T Lee, Michael G. Jameson, Paul Keall, Daniel Moses, Allan Fowler

**Affiliations:** 1 Department of Radiation Oncology, Calvary Mater Newcastle, Edith St, Waratah, NSW 2298 Australia; 2 Faculty of Medicine and Health, Sydney Medical School, The University of Sydney, Sydney, NSW Australia; 3 Department of Radiation Oncology, Cancer Therapy Centre, Liverpool Hospital, Liverpool, NSW Australia; 4 South Western Clinical School, School of Medicine, University of New South Wales, Sydney, NSW Australia; 5 GenesisCare, Sydney, NSW Australia; 6 Faculty of Medicine, Vincent’s Clinical School, University NSW, Sydney, Australia; 7 Ingham Institute of Applied Medical Research, Liverpool, NSW Australia; 8 Image X Institute, University of Sydney, Sydney, NSW Australia; 9 Graduate School of Biomedical Engineering, Faculty of Engineering, University of New South Wales, Sydney, Australia; 10 Department of Medical Imaging, Prince of Wales Hospital, Randwick, NSW Australia

**Keywords:** Head and neck, Neoplasms, Systematic review, Magnetic resonance imaging, Positron-emission tomography, Tomography, Recurrence, Response, Mid-treatment

## Abstract

**Background:**

To systematically review and meta-analyse the prognostic value of quantitative mid-treatment imaging biomarkers for predicting locoregional tumour control in patients undergoing definitive radiotherapy for mucosal head and neck squamous cell carcinoma.

**Main body:**

A systematic literature search (2005–2023) was conducted in PubMed, EMBASE, Scopus, and Cochrane databases according to a pre-registered PROSPERO protocol. Studies evaluating quantitative imaging features derived from CT, MRI, or PET during radiotherapy were included. Imaging features were grouped as baseline, absolute mid-treatment, or relative mid-treatment (delta) parameters. A random-effects meta-analysis was performed on studies reporting receiver operating characteristic (ROC)-based area under the curve (AUC) values. Forty-one studies encompassing 1654 patients were included. Seventeen studies (n = 612 patients) reported sufficient data for meta-analysis. The pooled AUC for relative mid-treatment parameters was 0.796 (95% CI: 0.762–0.831), demonstrating higher predictive performance than absolute mid-treatment parameters (AUC 0.686; 95% CI: 0.628–0.745). Baseline parameters showed moderate predictive ability (AUC 0.736; 95% CI: 0.688–0.785), and while numerically lower than relative mid-treatment parameters, this difference did not reach statistical significance. Diffusion-weighted MRI (ΔADCmean) and FDG-PET (ΔMTV, ΔTLG) emerged as the most consistently predictive modalities. Relative measures offer practical advantages, including internal self-normalisation and improved reproducibility across imaging platforms.

**Conclusions:**

Relative mid-treatment imaging biomarkers demonstrate superior predictive performance compared to baseline and absolute measures, supporting their potential role in adaptive radiotherapy strategies. Further prospective multi-centre studies with standardised imaging protocols and external validation are essential for clinical translation.

**Supplementary Information:**

The online version contains supplementary material available at 10.1186/s13550-026-01446-4.

## Background

Mucosal head and neck cancers account for 4—5% of all cancers worldwide with rising incidence due to factors such as tobacco and alcohol consumption, and human papillomavirus (HPV) infection [1]. Definitive radiotherapy remains a cornerstone of organ-preserving treatment for mucosal head and neck squamous cell carcinoma (HNSCC). Despite curative-intent treatment, locoregional tumour recurrence occurs in 15–50% of patients, contributing to significant morbidity and mortality [2, 3]. Moreover, among those who are cured, treatment is often associate^[i]^d with substantial acute and long-term toxicities. A reliable imaging biomarker capable of predicting treatment response early during therapy could facilitate personalised treatment strategies—enabling timely intensification (e.g., radiotherapy dose escalation, chemotherapy modification, or early salvage surgery) or de-escalation (e.g., radiotherapy dose or volume reduction, omission of chemotherapy).

Medical imaging such as computed tomography (CT), magnetic resonance imaging (MRI) and positron-emission tomography (PET) offer the potential to characterise tumour biology using quantitative measures that can act as an early surrogate marker for treatment response. Anatomical imaging (CT and MRI) can provide morphological information such as tumour volume and cellular density, while functional imaging (e.g., PET and advanced MRI techniques) can assess tumour physiology—including perfusion, proliferation, hypoxia, and metabolism—parameters that are closely linked to treatment resistance and adverse clinical outcomes [4]. For instance, ^18^F-fluorodeoxyglucose (FDG)-PET has the potential to assess tumour cell density, proliferation and cellular metabolism [5, 6]. Novel PET tracers such as ^18^F-fluromisonidazole (FMISO), nitroimidazole derivate ^18^F-fluoroerythronitroimidazole (FETNIM) and 2-nitroimidazole nucleoside analogue ^18^F-flortanidazole (FHX4) enable assessment of tumour hypoxia. Tumour cellular proliferation has been explored using thymidine analogue 3’-Deoxy-3’-^18^F-fluorothymidine (FLT) and 4’-methyl-^11^C-thiothymidine (4D-ST). Dynamic contrast-enhanced MRI (DCE-MRI) can provide information on tumour perfusion and permeability. Diffusion-weighted magnetic resonance imaging (DWI) depends on the microscopic mobility of water, which can be quantified using apparent diffusion coefficients (ADC) to measure tumour microenvironment and cellular density. The intra-voxel incoherent motion (IVIM) based on DWI technique, can be used to separate the signal for tissue perfusion from molecular diffusion to reflect tumour cellularity and vascularity. Imaging features that are extracted range from global measures (e.g., volume, maximum, mean) to higher order features that aim to map and quantify tumour heterogeneity. Published studies utilising only pre-treatment imaging for locoregional tumour response prediction have provided variable results [7, 8].

Imaging performed during treatment, referred to as mid-treatment imaging, has the potential to be a better biomarker of treatment response by capturing early biological response to delivered therapy [9, 10]. There has been a marked increase in studies evaluating the use of mid-treatment imaging in mucosal head and neck treatment during radiotherapy. Despite this interest, the results from mid-treatment imaging studies remain inconsistent, largely due to methodological heterogeneity in image acquisition, analysis, and reporting. A key area of variability lies in how mid-treatment imaging data are analysed and reported: some studies report static absolute imaging values at mid-treatment time points, while others report relative changes from baseline (i.e., delta-values). The use of absolute mid-treatment imaging values has the advantage of measuring tumour qualities of the residual tumour at a particular stage during treatment. Whereas, the use of relative mid-treatment imaging values has the advantage of measuring the change during treatment and hence sensitivity to prescribed treatment. Currently, there is no consensus on the optimal method for analysis of mid-treatment imaging biomarkers, and this lack of standardisation presents a barrier to the integration of mid-treatment imaging biomarkers into clinical decision-making.

The aim of this systematic review is to summarise the existing literature on the prognostic value of mid-treatment imaging during radiotherapy for mucosal HNSCC using locoregional recurrence as the primary clinical outcome. Additionally, we aim to identify the optimal method for analysis of mid-treatment imaging parameters for treatment response assessment.

## Main text

### Methods

A prospectively registered protocol is available through the International Prospective Register of Systematic Reviews (PROSPERO) (CRD42023484250). The review was conducted in accordance with the Preferred Reporting Items for Systematic Reviews and Meta-Analyses (PRISMA) guidelines [11].

#### Search strategy and study selection

A systematic search of PubMed, EMBASE, Scopus, and the Cochrane Central Register of Controlled Trials (CCTR) was conducted to identify relevant published studies between 2005 and 2023 (see Appendix, Supplementary Material). The PICOS framework (patient, intervention, comparison, outcome, and study) was used to develop the review question, focusing on the predictive value of quantitative mid-treatment imaging for locoregional treatment response in head and neck cancer treated with definitive radiotherapy. As outlined in the pre-specified protocol, exclusion criteria included: (1) studies without quantitative image analysis; (2) studies published prior to 2005; (3) non-squamous cell carcinoma (non-SCC) histology; (4) primary cutaneous or thyroid malignancies; and (5) studies with fewer than 10 patients. Additionally, studies primarily involving nasopharyngeal carcinoma (NPC) were excluded due to the distinct biological and clinical behaviour of NPC. Articles involving patients treated with adjuvant (post-operative) radiotherapy were also excluded.

Article screening, selection and data extraction were performed using the Covidence™ web-based systematic review platform. Two reviewers (YT, ML) independently reviewed titles and abstracts, with disagreements resolved by consensus or consultation with a third reviewer (MJ). In cases of overlapping patient populations, the study with the largest cohort was included.

#### Data extraction

The primary outcome for this review was locoregional disease control following completion of radiotherapy, considered the gold standard endpoint for treatment efficacy in this population.

Data on study characteristics (author, design, year, eligible patients, median follow-up), patient demographics (age, primary sub-site, tumour stage), treatment details (radiotherapy dose, concurrent chemotherapy), imaging protocol (modality, timing, region of interest, segmentation method), clinical outcomes and imaging parameters. Data were extracted by two independent reviewers and where information was missing, assumptions were not made and data were excluded from synthesis.

Imaging parameters were classified into types of features; global (i.e. volume, max, mean, median), histogram, textural and parametric map. Time of mid-treatment imaging was noted in weeks and further classified as early (week 1 to 3) or late (week 4 to 7).

#### Quality assessment

We assessed the risk of bias and applicability of the eligible studies using the Quality Assessment of Diagnostic Accuracy studies (QUADAS-2) tool [12]. This tool assesses four key domains: patient selection, index test, reference standard, and flow and timing, each of which is evaluated for risk of bias, while the first three are also assessed for concerns regarding applicability. Patient selection examines how participants were recruited and whether selection methods could introduce bias. The index test domain evaluates how the imaging was conducted and interpreted, including whether blinding or pre-specified thresholds were used. The reference standard assesses the reliability and appropriateness of the outcome measure used to determine true disease status. Flow and timing considers whether all patients received the same reference standard and whether there were any delays or losses to follow-up that could affect the results. Perceived quality were graded as low, high or unclear risk.

#### Data synthesis and meta-analysis

Imaging parameters extracted from baseline and mid-treatment imaging including absolute and/or delta-values (Δ, relative change in values acquired from imaging before and during treatment) were included in data synthesis. Imaging parameters were grouped into three categories for analysis: (1) baseline; (2) absolute mid-treatment; and (3) relative mid-treatment (delta) values.

For the meta-analysis, studies that performed receiver operating characteristics (ROC) analysis were selected. Imaging parameters were compared using the reported AUC values and standard errors from the ROC analysis in the MedCalc software. Heterogeneity across studies was quantified using the Higgins’ I^2^ statistic (I^2^ < 25%: negligible; 25–50%: low; 50–75%: moderate; > 75%: high heterogeneity), which estimates the proportion of variation due to heterogeneity rather than chance. A random-effects model was employed to evaluate AUC values and compare subgroups of baseline, absolute mid-treatment, and relative mid-treatment imaging parameters. Results were tabulated and visualised using forest plots and funnel plots to display individual study outcomes and pooled estimates.

## Results

### Study selection

**Fig. 1:**
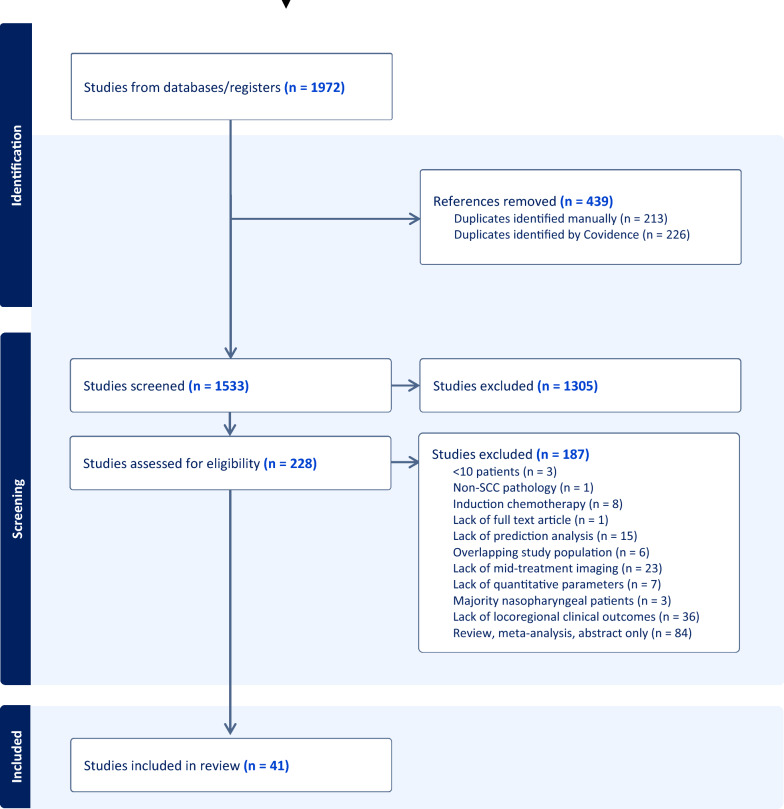
PRISMA flow diagram of included studies

A total of 1972 articles were identified through the search strategy. After removal of duplicates, 1,533 articles were screened. Full-text review was performed on 228 articles, resulting in 41 studies that met the inclusion and exclusion criteria. The study selection process and reasons for article exclusion are summarised in the PRISMA flowchart (Fig. 1). Common reasons for exclusion included lack of locoregional treatment outcomes, absence of mid-treatment imaging, lack of predictive or prognostic analysis, and review article format.

### Study characteristics

The total study population consisted of 1654 patients, of whom 82% received chemoradiotherapy and 18% received radiotherapy alone. Median follow-up ranged from 12 to 57 months, though not reported in five studies. The majority of studies were prospectively conducted (83%, 34 of 41). Twenty-seven studies used one imaging modality [13–39], 12 studies used two imaging modalities [40–51], and two studies used three imaging modalities [52, 53]. When reporting mid-treatment parameters, 21 (51%) studies reported relative values, five (12%) studies reported absolute values and 15 (37%) studies reported both absolute and relative values. There was also methodological heterogeneity, including differences in ROI selection, ROI delineation methods and timing of mid-treatment imaging. See summary of included studies (Tables 1 and 2).

**Table 1:** Summary of included studies of demographic, clinicopathological and treatment details

Author/ year	Journal	Study design	Year conducted	Median follow-up (months)	Eligible patients	Age (median)	Tumour site (%)	HPV positive	AJCC stage (%)	Treatment	Radiotherapy dose (median)	Chemotherapy details
Abramyuk et al. 2015	BJR	PS	2005—2006	24	15	50	OPSCC, HPX (%NR)	NR	2, 3, 4 (NIL %)	CRT (100%)	72GY/40#, BD	cisplatin, 5FU
Arens et al. 2014	EJNMMI	PS	2006—2008	52	46	60	OPSCC (52%), LARYNX (29%), HPX (17%), OC (2%)	NR	2 (25%), 3 (25%),4A (50%)	CRT (30%)	68GY/34#, 6#/week	cisplatin
Bhatia et al. 2010	BJR	PS	2001—2008	41	48	59	OPSCC (35%), HPX (35%), LARYNX (26%), NASAL (4%)	NR	3, 4 (NIL %)	CRT (100%)	72GY/40#, BD	cisplatin
Cao et al. 2019	FIO	PS	2014—2018	24	54	61	OPSCC (65%), HPX (11%), OC (11%), NPC (5%),SINONASAL (4%)	57%	3, 4 (NIL %)	CRT (100%)	70GY/35#; 80GY/35#,5#week	cisplatin, carboplatin
Castaldi et al. 2012	RAD ONC	PS	2007—2009	29	26	63	OPSCC (46%), HPX (35%), NPC (12%), LARYNX (7%)	NR	2 (4%), 3 (27%), 4 (69%)	CRT (100%)	78GY/65#, BD	cisplatin, cetuximab
Chen et al. 2014	LARYNGOSCOPE	PS	2009—2012	23	51	52	HPX (41%), OPSCC (39%), NPC (20%)	NR	3 (31%), 4A (69%)	CRT (86%)	72GY/40#, BD	cisplatin, cetuximab
El-Habashy et al. 2023	CTRO	PS	2019—2021	20	30	64	OPSS (70%), LARYNX *(23%), HPX (3%), UNPSCC (3%)	73%	1 (70%), 2 (13%),3 (10%), 4 (7%)	CRT (63%)	68GY/32#, 5#/week	NR
Farrag et al. 2009	NUC MED COMM	PS	2005—2008	13	43	56	HPX (35%), OPSCC (28%), OC (21%), NPC (9%), LARYNX (7%)	NR	NR	CRT (37%)	70GY/33#, 5#/week	cisplatin
Galban et al. 2009	TRANS ONC	PS	NR	NR	15	NR	OPSCC (80%), NP 7%), UPSCC (7%), HPX (7%)	NR	3 (53%), 4 (47%)	CRT (100%)	70GY/35#, 5#/week	cisplatin, carboplatin, taxol,5FU
Hentschel et al. 2011	EJNMMI	PS	2005—2009	26	37	55	OPSCC (49%), HPX (32%), OC (11%), LARYNX (8%)	NR	NR	CRT (100%)	72GY/40#, BD	cisplatin, 5FU
Hoeben et al. 2013	JNM	PS	2006—2008	32	48	60	OPSCC (54%), LARYNX (28%), HPX (16%), OC (2%)	NR	2 (24%), 3 (36%),4 (50%)	CRT (30%)	68GY/34#, 6#/week	cisplatin
Hu et al. 2019	MEDICINE	PS	2006—2012	54	32	NR	LARYNX (41%), HPX (34%), OPSCC (16%), OC (9%)	NR	NR	CRT (100%)	70GY/35#, 5#/week	cisplatin
Kabarriti et al. 2018	IJROBP	RS	2007—2015	34	96	64	OPSCC (100%)	57%	2 (9%), 3 (17%),4 (74%)	CRT (80%)	70GY/33#, 5#/week	cisplatin, cetuximab, carboplatin
Khattab et al. 2020	EJRNM	PS	2017—2019	NR	46	51	LARYNX (35%), HPX (22%), NPX (20%), OC (15%), SINUS (7%), OPSCC (2%)	NR	2 (4%), 3 (24%), 4 (72%)	CRT (84%)	70GY/33#, 5#/week	cisplatin
Kim et al. 2009	CCR	PS	2005—2007	12	33	61	OPSCC (100%)	NR	4 (100%)	CRT (79%)	70GY/32#, 6#/week	cisplatin, cetuximab
King et al. 2013	RADIOLOGY	PS	2004—2008	44	37	57	OC/OPSCC (38%), HYPX/LARYNX (54%), NASAL/SINUS (8%)	NR	2 (24%), 3 (22%), 4 (54%)	CRT (89%)	NR	NR
King et al. 2010	JMRI	PS	NR	39	47	57	OPSCC/OC (47%), HYX/LARYNX (43%), NPC (6%), OTHER (4%)	NR	NR	CRT (91%)	NR	NR
Lafata et al. 2021	MED PHYS	PS	2012—2016	47	64	59	OPSCC (100%)	89%	NR	NR	70GY/33#, 5#/week	cisplatin, docetaxel
Lambrecht et al. 2014	RAD ONC	PS	2004—2009	57	20	NR	OPSCC (55%), HPX (30%), LARYNX (15%)	NR	4 (100%)	CRT (95%)	70GY/33#, 5#/week	NR
Lazzeroni et al. 2021	PIRO	PS	NR	20	28	60	OPSCC (32%), HPX (29%), LARYNX 14%), OC (7%)	79%	NR	CRT (100%)	70GY/33#, 5#/week	cisplatin
Lee et al. 2018	APJCO	RS	2009—2015	31	35	59	OPSCC (100%)	29%	2 (14%), 3 (23%), 4 (63%)	CRT (100%)	72GY/40#, BD; 70GY/32#, 5#/week	cisplatin, 5FU, docetaxel, cetuximab
Lin et al. 2021	CLIN ONC	RS	2009—2015	40	46	55	OPSCC (100%)	100%	2 (4%), 3 (20%), 4 (76%)	CRT (100%)	70GY/35#, 5#/week	cisplatin, cetuximab
Min et al. 2015	EJNMMI	RS	2009—2014	25	75	60	OPSCC (65%), LARYNX (22%), HPX (8%), OC (4%)	NR	3 (25%), 4 (75%)	CRT (79%)	70GY/35#, 5#/week	cisplatin, cetuximab, carboplain, 5FU
Martens et al. 2022	CANCERS	PS	2013—2019	31	57	63	OPSCC (79%), HPX (21%)	44%	3 (35%), 4 (65%)	CRT (93%)	70GY/33#, 5#/week	cisplatin, cetuximab
Marzi et al. 2017	EUR RAD	PS	2010—2013	28	34	55	OPSCC (41%), NPC (38%), HPX (18%), UNPSCC (2%)	NR	NR	CRT (100%)	70GY/33#, 5#/week	cisplatin
Matoba et al. 2014	AJNR	PS	2008—2012	31	35	66	HPX (34%), LARYNX (29%), OPSCC (26%), OC (11%)	NR	NR	CRT (100%)	70GY/35#, 5#/week	cisplatin, S-1
Mishra et al. 2013	JMIRO	RS	2009—2011	18	38	58	OPSCC (76%), LARYNX (16%), OTHER (3%)	NR	1 (14%), 2 (46%), 3 (24%), 4 (16%)	CRT (93%)	70GY/35#, 5#/week	cisplatin, cetuximab
Mitamura et al. 2021	EJNMMI	PS	2011—2020	NR	32	63	HPX (52%), OPSCC (25%), LARYNX (16%), OC (6%), NPC (3%)	NR	NR	CRT (100%)	70GY/35#, 5#/week	cisplatin, 5FU, S-1, docetaxel
Mohamed et al. 2022	RAD ONC	PS	2017—2021	31	81	61	OPSCC (83%), UNPSCC (15%), UNKNOWN (2%)	90%	1 (47%), 2 (25%), 3 (21%), 4 (7%)	CRT (80%)	70GY/35#, 5#/week	NR
Morgan et al. 2021	QIMS	RS	2014—2019	14	90	NR	OPSCC (62%), HPX (31%), LARYNX (7%)	47%	NR	CRT (98%)	70GY/33#, 5#/week	cisplatin, cetuximab, carboplatin
Sanduleanu et al. 2020	CTRO	PS	NR	26	33	60	OPSCC (50%), LARYNX (29%), HPX (18%), OC (3%)	32%	NR	CRT (67%)	70GY/35#, 5#/week	cisplatin, cetuximab
Scalco et al. 2016	PHY MED	PS	2011—2014	27	30	58	OPSCC (40%), NPC (37%), HPX (17%), LARYNX (3%),	NR	NR	CRT (100%)	70GY/33#, 5#/week	cisplatin
							UNPCC (3%)					
Tomita et al. 2022	EUR RAD	RS	NR	20	70	69	HYPX (61%), LARYNX (39%)	NR	1 (1%), 2 (20%),3 (21%), 4 (57%)	CRT (90%)	70GY/35#, 5#/week	cisplatin, docetaxel, cetuximab, S-1, 5FU
Trada et al. 2023	EUR RAD	PS	2014—2019	34	55	61	OPSCC (67%), HPX (13%), LARYNX (13%), NPC (7%)	34%	2 (11%), 3 (26%), 4 (64%)	CRT (84%)	70GY/35#, 5#/week	cisplatin, cetuximab, carboplatin
Trada et al. 2023	QIMR	PS	2014—2019	31	52	62	OPSCC (75%), HPX (13%), LARYNX (11%)	37%	2 (11%), 3 (24%), 4 (52%)	CRT (84%)	70GY/35#, 5#/week	cisplatin, cetuximab, carboplatin
Truong et al. 2011	AJNR	PS	2007—2008	28	15	58	OPSCC (33%), LARYNX (20%), HPX (13%), NPC (13%), OC (7%), UNPSCC (7%)	NR	2 (13%), 3 (20%), 4 (67%)	CRT (87%)	70GY/35#, 5#/week	cisplatin, cetuximab
Ursino et al. 2016	BJR	PS	2012—2015	NR	25	NR	OPSCC (28%), NPC (24%), LARYNX (20%), OC (20%), HPX (8%)	NR	3 (36%), 4 (64%)	CRT (100%)	66GY/30#, 5#/week	cisplatin
Vandecaveye et al. 2010	EUR RAD	PS	NR	NR	30	53	HPX (50%), OPSCC (43%), LARYNX (7%)	NR	1 (3%), 3 (23%), 4 (73%)	CRT (90%)	72GY/40#, BD	NR
Wang et al. 2012	MED PHYS	PS	NR	20	14	58	OPSCC (64%), HPX (14%), NPC (7%), UNPSCC (7%)	NR	4 (100%)	CRT (100%)	70GY/35#, 5#/week	carboplatin, paclitaxel, cisplatin, cetuximab
Wiedenmann et al. 2015	RAD ONC	PS	NR	44	16	NR	NR	NR	NR	CRT (100%)	70GY/33#, 5#/week	cisplatin
Wong et al. 2018	ENJMMI	PS	2014—2016	14	35	61	OPSCC (83%), HPX/LARYNX (17%)	63%	NR	CRT (100%)	65GY/30#, 5#/week	cisplatin, cetuximab, carboplatin

**Table 2:** Summary of included studies of imaging modalities, technique, ROI segmentation and feature extraction

Author/ year	Eligible patients	Imaging modality	Imaging technique	Imaging timepoints	ROI	ROI segmentation	Extracted parameters	Type of mid- treatment measurement	Outcome	Predictive parameter summary
Abramyuk et al. 2015	15	CT	DCE CT	DCE-CT: W0, W2, W5	PT	Manual	Global: Ktrans, rTBV Histogram: lacunarity	Relative	LRR	NS: "no correlation to 24 m tumour behaviour". Qualitative analysis—increase in slope of ktrans and rTBV was present in good responders
Arens et al. 2014	46	PET, CT	FLT	FLT-PET/CT: W0, W2, W4	PT	CT: manual. FLT: manual, semi-automated (threshold, gradient, TBR)	Global: volume	Absolute, Relative	LRR	Only ∆W4 FLT-PET volume using FLAB technique correlated to RFS. No FLT-PET or CT measures correlated to LRR
Bhatia et al. 2010	48	MRI	T1 GAD	MRI: W0, W2	PT	Manual	Global: volume	Absolute, Relative	LF	W0 MRI volume, W2 volume, ∆W2 volume predicted for 12 m LF
Cao et al. 2019	54	MRI	DCE-MRI, DWI	DWI/ DCE-MRI: W0, W2	CM	Manual	Global: mean, volume	Absolute, Relative	LF	W2 tumour blood volume correlated to DM
Castaldi et al. 2012	26	PET	FDG	FDG-PET: W0, W2	CM	Manual	Global: max	Relative	LRR	Change in SUVmax at mid-treatment did not correlate to LRR
Chen et al. 2014	51	PET, CT	FDG, CT	FDG-PET/CT: W0, W4—5	PT, IN	Manual	Global: max, volume	Absolute, Relative	LF	∆W2 SUVmax correlated to LF
El-Habashy et al. 2023	30	MRI	DWI	DWI: W0, W1, W2, W3, W4, W5, W6, W7	PT, IN	Manual	Global: mean. Histogram	Relative	LRR	Nil ADC parameters correlated to clinical outcomes following radiotherapy
Farrag et al. 2009	43	PET	FDG	FDG-PET: W0, W4	CM	Manual	Global: max	Absolute	LRR	Mid-treatment SUVmax correlated to OS but not LRR
Galban et al. 2009	15	MRI	DWI	DWI: W0, W3	CM	Manual	Global: max, volume. PRM	Relative	LRR	∆W3 PRM ADC correlated to disease progression at 6 months
Hentschel et al. 2011	37	PET	FDG	FDG-PET/CT: W0, W1, W3, W5	CM	Semi-automated (TBR)	Global: max, mean, volume	Relative	LRR	∆W2 SUVmean > 40% correlated to LRR
Hoeben et al. 2013	48	PET	FLT	FLT-PET: W0, W2, W4	PT	Manual	Global: max, volume	Relative	LRR	∆W4 FLT-PET volume (visual) and ∆W2 SUVmax correlated to LRR
Hu et al. 2019	32	PET	FETNIM	FETNIM-PET:W0, W5	PT, IN	Manual	Global: max	Absolute	LRR	W0 SUVmax predict OS. Nil correlations to LRR
Kabarriti et al. 2018	96	CT	CT	CT: W0, W3	CM	Manual	Global: volume	Relative	LRR	∆W3 CT-GTV correlated to LRR
Khattab et al. 2020	46	MRI	DWI	DWI: W0, W2—3	PT	Manual	Global: mean, volume. Histogram	Relative	LF	Pretreatment ADCmean and ∆ADCmean correlated to LF
Kim et al. 2009	33	MRI	DWI	DWI: W0, W1	TN	Manual	Global: mean, volume	Relative	LRR	Pretreatment ADCmedian and ∆ADCmedian correlated to LRR
King et al. 2013	37	MRI	DWI	DWI: W0, W2	PT	Manual	Global: mean. Histogram	Relative	LF	Mid-treatmetn ADCmean, kurtosis and skewness correlated to LF
King et al. 2010	47	MRI	MRS	MRS: W0, W2	PT	Manual	Global: CHO ratio	Relative	LRR	No correlation was found between MRS and LRR
Lafata et al. 2021	64	PET	FDG	FDG-PET: W0, W2	PT	Manual	Textural features	Absolute	LRR	Intra-treatment radiomic features correlted to RFS
Lambrecht et al. 2014	20	MRI	DWI	DWI: W0, W2	CM	Manual	Global: mean	Relative	LRR	Change in mid-treatment ADCmean correlated to LRR
Lazzeroni et al. 2021	28	PET	FMISO	F-MISO: W0, W2, W5	CM	Semi-automated (threshold)	Global: volume	Absolute, Relative	LRR	Mid-treatment change in hypoxic volume correlated to LRR
Lee et al. 2018	35	CT	CT	CT: W0, W4	PT	Manual	Global: volume	Absolute, Relative	LF	Mid-treatment CT GTV volume correlated to 2—yr LF
Lin et al. 2021	46	PET	FDG	FDG-PET: W0, W3	TN	Semi-automated (threshold)	Global: max, mean, volume, TLG	Absolute, Relative	LRR	50% reduction in TN correlated to LRR
Min et al. 2015	75	PET	FDG	FDG-PET: W0, W3	PT	Semi-automated (threshold)	Global: max, mean, volume, TLG	Absolute, Relative	LRR	W3 TLG correlated to LRR
Martens et al. 2022	57	PET, MRI	FDG, DWI, T1 GAD	DWI/ DCE-MRI/ FDG-PET: W0, W2	PT	T1Gad/DWI: manual. FDG: semi-automated (threshold)	Global: median, peak, mean, TLG. Histogram	Absolute, Relative	LRR	Baseline and mid-treatment MR features correlated to LRR
Marzi et al. 2017	34	MRI	IVIM DWI	DWI: W0, W3	IN	Manual	Global: mean	Absolute, Relative	LRR	Baseline D, mid-treatment D and Δf correlated to NR
Matoba et al. 2014	35	MRI	DWI	DWI: W0, W3	CM	Manual	Global: mean, volume	Relative	LRR	Relative change in ADCmean correlated to LRR
Mishra et al. 2013	38	CT	CT	CT: W0, W1, W2, W3, W4, W4, W5, W6, W7	TN	Manual	Global: volume	Absolute, Relative	RF	lymph node volume did not correlate to NR
Mitamura et al. 2021	32	PET	FDG, 4DST	FDG / 4DST PET: W0, W4	PT	FDG/4DST: semi-automated (threshold)	Global: max, volume	Relative	LRR	Change in primary tumour volume measured using 4DST-PET correlated to residual disease
Mohamed et al. 2022	81	MRI	DWI	DWI: W0, W3—4	CM	Manual	Global: mean. Histogram	Relative	LRR	ΔW ADCmean correlated to LC and DFS
Morgan et al. 2021	90	CT	CT	CT: W0, W4	CM	Manual	Textural features	Relative	LRR	Baseline and mid-treatment cluster of radiomic features correlates to LRR
Sanduleanu et al. 2020	33	PET	FHX4	FHX4-PET: W0, W2	PT	Manual, semi-automated (TBR)	Global: volume	Absolute, Relative	RF	Change in hypoxia volume during radiotherapy correlated to RFS
Scalco et al. 2016	30	MRI	DWI, T2	DWI, T2: W0, W3	IN	Manual	Textural features. Global: mean	Relative	RF	Baseline ADC features correlated to NR
Tomita et al. 2022	70	MRI	DWI	DWI: W0, W4	PT	Manual	DL imaging features	Absolute	LR	Deep learning imaging features from intra-treatment DWI was correlated to LR
Trada et al. 2023	55	PET, MRI	FDG, DWI	DWI: W0, W2, W3, W5, W6 FDG-PET: W0, W3	PT	DWI: manual. FDG: semi-automated (gradient)	Global: max, mean, volume	Absolute, Relative	LR	Change in ADCmean and MTV correlated to local recurrence
Trada et al. 2023	52	PET, CT	FDG, CT	FDG-PET/ CT: W0, W3	PT	CT: manual. FDG: semi-automated (gradient, threshold)	Global: volume	Relative	LRR	Change in MTV but not CT volume correlated to LRR
Truong et al. 2011	15	CT	DCE CT	CT: W0, W2, W4, W6	PT	Manual	Global: blood flow, blood volume, mean transit time, CT perfusion	Absolute, Relative	LRR	Increase in blood flow correlated to LRR
Ursino et al. 2016	25	CT	DCE CT	CT: W0, W3	NR	Manual	Global: blood flow, blood volume, mean transit time, CT perfusion	Relative	LRR	Change in blood flow and volume correlated to early FDG-PET response
Vandecaveye et al. 2010	30	MRI	DWI	DWI: W0, W2, W4	CM	Manual	Global: mean, volume	Relative	LRR	Change in ADCmean but not change in volume correlated to 2yr LRR
Wang et al. 2012	14	MRI	DCE-MRI	DCE-MRI: W0, W2	CM	Manual, automatic clustering	Global: blood flow, blood volume	Relative	LRR	Mid-treatment blood volume correlated to LR
Wiedenmann et al. 2015	16	PET	FMISO	F-MISO: W0, W2, W5	CM	Semi-automated (threshold)	Global: max, volume	Absolute	LRR	Mid-treatment TBRmax correlated to tumour recurrence
Wong et al. 2018	35	PET, MRI	FDG, DWI, DCE	FDG-PET/ DWI/ DCE: W0, W1, W2	CM	DCE/DWI: manual. FDG: semi-automated (threshold)	Global: mean, volume, blood flow, blood volume, Ktrans, max, TLG	Relative	LRR	Change in TLG, SUVmax at week 1 correlated to DFS. Change in ADC, Ktrans and V3 at week 2 correlated to DFS. ΔADC best correlated to DFS

MRI imaging was utilised in 19 studies, PET imaging in 19 studies and CT imaging in 9 studies. DWI-MRI was the most common mid-treatment imaging modality reported (15 studies), followed by FDG-PET (12 studies).(Table 3).

**Table 3:** Overview of included studies

			**Patients**		**Segmentation method**		**Parameters types extracted**			
	Number of studies	Follow-up months (mean)	Number of patients (Total)	Age (mean)	Manual	Semi-automated	Global	Histogram	Textural	Parametric map
**CT**	9		357		9 (100%)	0 (0%)				
Anatomical	6	27	309	59	6 (100%)	0 (0%)	6	0	1	0
DCE-CT	3	24	55	50	3 (100%)	0 (0%)	2	1	0	0
**MRI**	19		771		19 (100%)	0 (0%)				
Anatomical	7	29	260	60	7 (100%)	0 (0%)	6	0	1	0
DWI	15	27	662	61	15 (100%)	0 (0%)	13	4	2	1
DCE-MRI	4	21	160	60	4(100%)	0 (0%)	4	0	0	0
MRS	1	39	47	57	1 (100%)	0 (0%)	1	0	0	0
**PET**	19		756		6 (32%)	13 (68%)				
FDG	12	28	573	59	4 (33%)	8 (67%)	12	0	1	0
F-MISO	2	32	44	60	0 (0%)	2 (100%)	2	0	0	0
FLT	2	42	94	60	1 (50%)	1 (50%)	2	0	0	0
FHX4	1	26	33	60	0 (0%)	1 (100%)	1	0	0	0
4D-ST PET	1	NR	32	63	0 (0%)	1 (100%)	1	0	0	0
FETNIM	1	54	32	NR	1 (100%)	0 (0%)	1	0	0	0

### Study quality

**Fig. 2:**
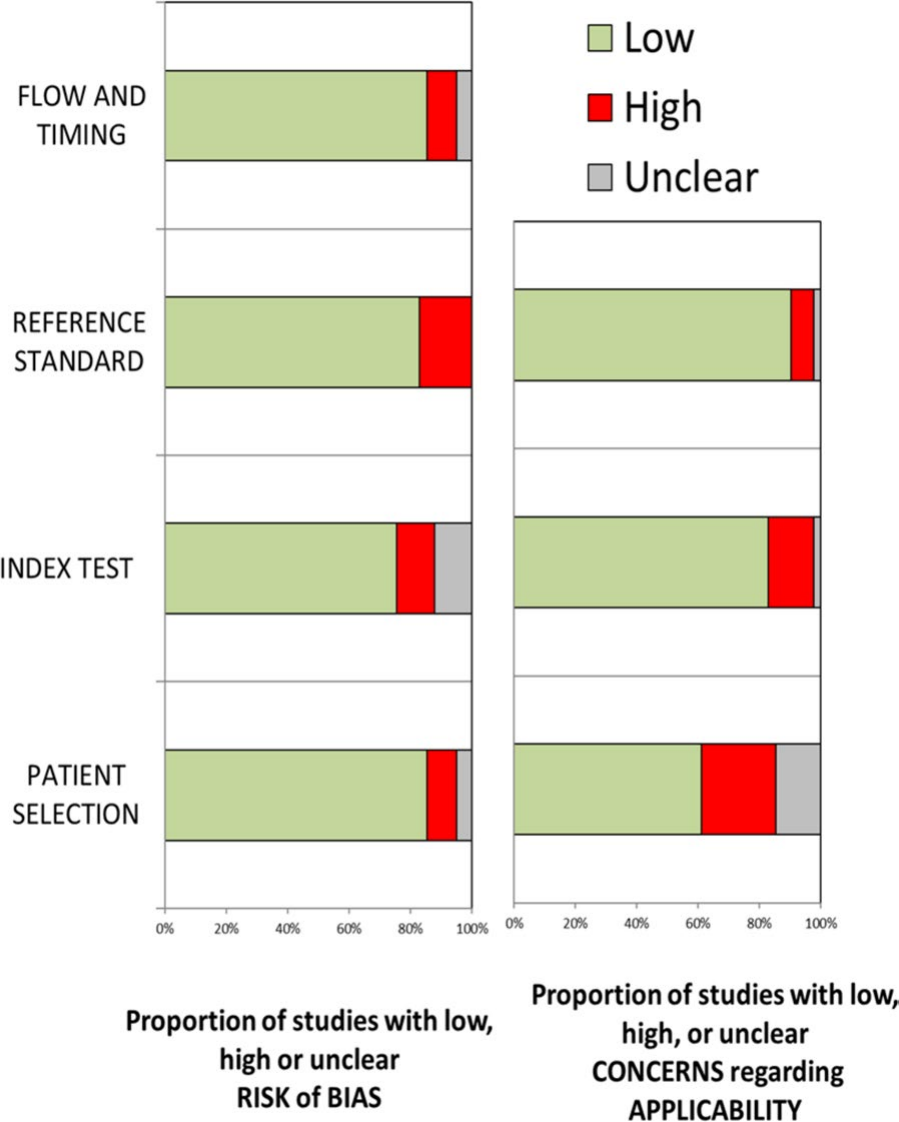
Risk of bias and applicability of the eligible studies using the quality assessment of diagnostic accuracy studies (QUADAS-2) tool

Study quality was assessed using the QUADAS-2 tool. The risk of bias was low across the four domains with 85%, 76%, 83% and 85% of studies having low risk in the patient selection, index test, reference standard and timing domains, respectively.(Fig. 2) Overall, 14 out of 41 studies (34%) demonstrated high risk of bias in at least one of the domains. Reference standard domain posed the highest risk of bias (17%, 7 of 41 studies).

Concern regarding applicability was generally low for most studies with 61%, 83% and 90% of studies having low concerns of applicability in the patient selection, index test and reference standard domains, respectively (Fig. 2). Overall, 16 out of 41 studies (39%) demonstrated high concerns regarding applicability in at least one of the domains. Patient selection domain posed the highest concern regarding applicability (24%, 10 of 41 studies).

### CT studies

CT was used in nine studies involving 357 patients.(Table 3) Anatomical CT was the most common modality analysed (6 of 9 studies) [22, 28, 32, 40, 42, 49], with all studies employing manual ROI segmentation. Global parameters, particularly tumour volume, were most frequently analysed (6 of 7 studies). A range of relative change in tumour volumes were noted on mid-treatment imaging (median 19—68%) due to methodological variability in choice of ROI and timing of mid-treatment imaging. Three studies reported statistically significant correlations [22, 28, 42], and three studies did not find any correlation between mid-treatment CT volume and clinical outcomes [32, 40, 49]. The largest CT study by Kabarriti et al. (n = 96 patients) found a relative change in CT tumour volume (cut-off Δ19%, HR 0.26) but not absolute CT tumour volume at week 3 correlated to locoregional outcomes [22]. Similarly, Lee et al. reported that both absolute volume (11 cc, AUC 0.847) and relative change in volume (Δ75%, AUC 0.725) at week 4 mid-treatment CT were predictive for locoregional outcomes [28]. Conversely, studies by Arens et al., Mishra et al. and Trada et al. did not find any correlation between change in CT based tumour volume and clinical outcomes [32, 40, 49]. Morgan et al. conducted a matched case–control study (n = 90 patients) using textural analysis and found that relative change in a cluster of mid-treatment CT features were associated with locoregional recurrence [34].

Three studies used quantitative contrast enhanced CT [13, 37, 38], all employing manual ROI segmentation and primarily analysing global features (2 of 3 studies). Studies by Troung et al. and Ursino et al. reported multiple mid-treatment perfusion characteristics. Troung et al. (n = 15 patients) found that both absolute value (136 vs 44 ml/100 g/min) and relative change (Δ, 28% vs 18%) in mid-treatment blood flow measurements at week 2 were higher in patients with tumour control [37]. Ursino et al. (n = 25 patients) found relative change in blood volume, blood flow and permeability surface measurements at week 3 correlated to early FDG-PET tumour response [38]. Abramyuk et al. (n = 15 patients) measured multiple perfusion characteristics at week 2 and week 5 during radiotherapy but did not find correlation to tumour outcomes [13].

### PET studies

PET was utilised in 19 studies containing 756 patients. (Table 3) FDG-PET was the most frequent PET tracer based imaging analysed (12 of 19 studies) [15, 17, 19, 25, 29, 30, 42, 46, 48, 49, 52, 53]. Other novel hypoxia PET tracers based imaging utilised included F-MISO (2 studies) [27, 39], FHX4 (1 study) [35], and FETNIM (1 study)[21]. Novel tumour proliferation PET tracers utilised included FLT PET (2 studies) and 4D-ST PET (1 study) [46]. An advantage of PET imaging is relative ease of implementation of semi-automated segmentation methods for ROI delineation which was used in majority of PET studies (12 of 19). Global features such as maximum voxel value (SUV_max_), metabolic tumour volume (MTV) and total lesional glycolysis (TLG) were the commonly utilised (19 studies). Textural features were analysed in only 1 study [25].

FDG-PET was the most utilised PET tracer due to its relative ease of access from its current clinical use in diagnosis, staging and post treatment monitoring in management of head and neck malignancies. The majority of FDG-PET studies performed imaging early (week 1—3) during radiotherapy (9 of 12 studies). Overall, there were nine FDG studies [19, 25, 29, 30, 42, 48, 49, 52, 53] that found a correlation between mid-treatment imaging features and clinical outcomes and three studies [15, 17, 46] which did not. A study by Min et al., utilised week 3 FDG PET in 75 patients during radiotherapy and analysed a range of absolute and relative mid-treatment global features from primary tumours [30]. They found absolute mid-treatment values of SUV_max_ (cut-off value 4.3 g/mL, 2 yr LRRFS 89% vs 75%, HR 3.7, p = 0.03), MTV (cut-off value 3.3cm^3^, 2 yr LRRFS 90% vs 69%, HR 3.9, p = 0.02) and TLG (cut off value 9.4 g, 2 yr LRRFS 93% vs 71%, HR 6.3, p < 0.01) correlated to locoregional recurrence. Of the studies that compared the performance of multiple mid-treatment global features, relative change in MTV and its closely related TLG were frequently found to be the best predictor of tumour control (Lin et al., ΔMTV 50%, HR 0.11, Trada et al., ΔMTV AUC 0.833; Trada et al., ΔMTV AUC 0.761; Wong et al. ΔTLG 0.825) [29, 48, 49, 53]. Lafata et al. performed textural analysis from week 2 FDG-PET in 64 patients with oropharyngeal malignancies [25]. They utilised an in-house software to analyse multiple radiomic features and unsupervised learning to identify three intra-treatment imaging clusters. They found a difference between the intra-treatment clusters and tumour recurrence (HR 10.5, p < 0.01).

Four studies utilised hypoxia PET tracers and analysed global imaging parameters such as SUV_max_ or hypoxic volume for treatment response prediction, with moderate success. Two studies employed first generation hypoxia tracer, F-MISO PET to correlate changes in hypoxia during radiotherapy with tumour recurrence [27, 39]. Lazzeroni et al. utilised a volumetric measure of hypoxia sub-volume (%HTV) and found that a relative change (AUC 0.75, cut-off value < 44.3%), but not absolute HTV value at week 2 correlated to locoregional recurrence [27]. Wiedenman et al. utilised a modified hypoxia SUV_max_ value (TBR_max_) and found that patients with local tumour recurrence had higher absolute TBR_max_ values at week 2 (p = 0.031) but not week 5 (p = 0.071) [39]. Alternate hydrophilic hypoxia PET tracers such as FHX4 and FETNIM with lower background tissue uptake were developed to improve the tumour to background signal and improve clearance with favourable pharmacokinetic properties. A study by Sanduleanu et al., involving 34 patients, found that relative residual hypoxic volume at week 2 on FHX4 PET correlated to local control (p = 0.028) [35]. Hu et al. reported a decrease in SUV_max_ at week 5 during treatment on FETNIM PET, but no significant correlation to local tumour control [21].

Two studies analysed FLT-PET to investigate the correlation between tumour proliferation and treatment response [20, 40]. A study by Hoeben et al. found a decrease in tumour volume and SUVmax during radiotherapy [20]. They found relative change in proliferative tumour volume (cut-off value > 78%, 3 yr LRC 100% vs 68%, p = 0.021) at week 4 correlated to locoregional outcomes. They did not find a correlation between relative change in SUVmax to outcomes. A study by Arens et al. employed variety of semi-automated methods for measuring proliferative tumour volumes but found no correlation to locoregional outcomes at either week 2 or week 4 during radiotherapy [40]. A study by Mitamura et al. utilised 4D-ST, a cell proliferation tracer that is resistant to thymidine phosphorylase degradation, and performed mid-treatment imaging at week 4 to calculate SUV_max_, proliferation tumour volume (PTV) and total lesional proliferation (TLP) [46]. They found change in tumour volume (ΔPTV AUC 0.91, cut-off value –90%), total proliferation (ΔTLP AUC 0.89, cut-off value –45%) and maximum voxel volume (ΔSUV_max_ AUC 0.75, cut-off value –99%) discriminated early treatment response.

### MRI studies

MRI was utilised in 19 studies containing 771 patients (Table 3). DWI MRI was the most common modality analysed (15 of 19 studies) [16, 18, 23, 26, 31, 33, 36, 43–45, 47, 48, 50, 52, 53]. Other MRI modalities utilised included anatomical MRI (7 studies)[14, 41, 43–45, 47, 51], DCE-MRI (4 studies) [41, 51–53], and MR spectroscopy (1 study) [24]. All 19 MRI studies utilised manual segmentation for ROI delineation. Global imaging features were again the most commonly parameters utilised (13 studies); however, several studies implemented histogram analysis (5 studies), textural features (2 studies) and parametric maps (1 studies).

All seven anatomical MRI imaging utilised tumour volume, while one study also utilised textural features for response assessment [47]. There were four studies that found statistically significant correlations [14, 43, 45, 47], and three studies that did not find any correlation between mid-treatment MRI volume and clinical outcomes [41, 44, 51]. Study by Bhatia et al. correlated primary tumour volume delineated on T1 MRI during week 2 of radiotherapy to clinical outcomes [14]. They found that absolute change in tumour volume (AUC 0.721, cut-off > 10.6 cc) had stronger correlation compared to relative change in tumour volume (AUC 0.689, cut-off < 9.4%) to local failure. A similar study by Khattab et al. also found that absolute primary tumour volume during week 2—3 of radiotherapy correlated to local failure (median 2.5 cc vs 14.9 cc, p = 0.007) [43]. They found that relative change in tumour volume did not reach statistical significance for local failure in their patient population (median 73% vs 35%, p = 0.100). In contrast, the largest series by Cao et al., containing 54 patients did not find a correlation between absolute or relative mid-treatment anatomical tumour volume on T1 MRI imaging and tumour control [41]. Study by Scalco et al. utilised higher order radiomics and found relative change in week 3 mid-treatment textural features (Variance, Accuracy 68%; Fractional dimension, Accuracy 64%) on T2 imaging correlated to tumour recurrence [47].

The majority of studies utilising DWI MRI imaging analysed global features (13 of 15 studies), predominantly mean apparent diffusion coefficient value (ADC_mean_). Tumour volume based on ADC maps was only evaluated in three studies with no correlation found with locoregional clinical outcomes [18, 50, 52]. The largest DWI series containing 81 patients by Mohamed et al., found relative change in primary tumour ADCmean at week 3 had the strongest correlation to local tumour control (cut-off < 7%, 2 yr LC 28% vs 96%) [33]. A study by Trada et al. analysed primary tumour ADC_mean_ at multiple time-points and found that relative change but not absolute mid-treatment ADC_mean_ correlated to local tumour control [48]. Relative change in primary tumour ADC_mean_ is a promising imaging biomarker for treatment response prediction. Relative change in ADC_mean_ with optimal cut-off (OC) values ranging from 16 to 33% (ΔADC_mean_) was consistently found to have strong correlation to clinical outcomes in studies by Khattab et al. (AUC 0.786, OC 33%), Kim et al. (AUC 0.88), Marzi et al. (AUC 0.662, OC 16%), Matoba et al. (AUC 0.900, OC 24%), Trada et al. (AUC 0.825, OC 24%), Vandecaveye et al. (AUC 0.97, OC 25%), and Wong et al. (AUC 0.937, OC 17%) [31, 43–45, 48, 50, 53]. Due to the image resolution of ADC maps derived from DWI MRI it is possible to derive histogram and higher order imaging features to quantitatively assess microenvironment and physiological processes for tumour response prediction. Histogram analysis was utlised in four studies [16, 23, 43, 52], higher order textural features were utilised in two studies [36, 47], and parametric maps were analysed in one study [18]. A study by Tomita et al. demonstrated the feasibility of applying artificial intelligence, using deep learning of mid-treatment DWI maps for imaging feature extraction, which correlated with local tumour recurrence (AUC 0.767) [36].

Intravoxel incoherent motion diffusion-weighted imaging (IVIM-DWI) MRI can be used to assess both water diffusion and microcirculation (perfusion) within tissues. It leverages the principle of DWI, but uses lower b-values to specifically isolate and quantify perfusion information. Two studies utlised IVIM for tumour response prediction. Study by Marzi et al. found that absolute mid-treatment diffusion co-efficient (D, AUC 0.75), perfusion co-efficient (D*, AUC 0.688) and perfusion fraction (f, AUC 0.829) correlated to nodal recurrence. Whereas Martens et al., did not find statistically significant correlations between these imaging features and clinical outcomes.

DCE-MRI is a complex MRI technique that analyses tissue enhancement patterns after the injection of a contrast agent to assess blood flow, vessel permeability, and tissue volume fractions. It provides functional information about tissue perfusion and microcirculation, particularly in tumours that can be used for treatment response assessment. DCE-MRI was analysed in four studies with moderate success utilising mid-treatment imaging features such as K_trans_ (volume transfer constant), V_e_ (fractional volume of extravascular extracellular space), K_ep_ (transfer rate of contrast agent from extravascular extracellular space to plasma [41, 51–53]. A study by Wong et al. found relative change in K_trans_ at week 2 had the highest correlation to clinical response (AUC 0.813) [53]. Similarly a study by Martens et al., found relative change in K_ep_, and absolute mid-treatment V_e_, K_trans_ and K_ep_ correlated to tumour outcomes [52].

Magnetic Resonance Spectroscopy (MRS) is a technique used alongside MRI to analyse the chemical composition of tissues. MRS focuses on identifying and quantifying specific metabolites within a selected region, studied most commonly in brain tumours. King et al. utlised a novel MRS technique to measure changes in mid-treatment tumoral metabolite Choline in patients with head and neck cancer. They detected changes in Choline, Choline:Creatinine and Choline:Water ratios during week 2 of radiotherapy but these did not correlate with clinical outcomes in their patient population.

### Meta-analysis

Seventeen studies containing 612 patients reported ROC analysis and were included in the meta-analysis. A total of 63 imaging parameters were evaluated: 17 baseline parameters, and 46 mid-treatment parameters (37 relative; 9 absolute).

**Fig. 3:**
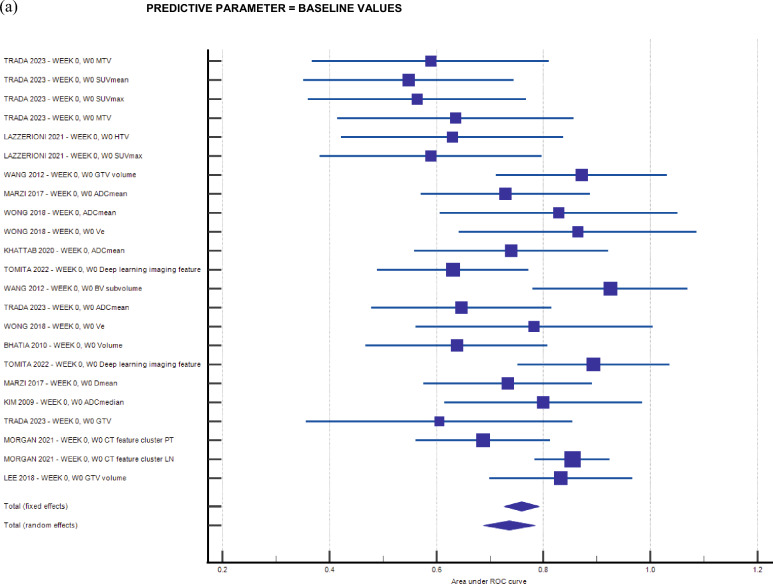

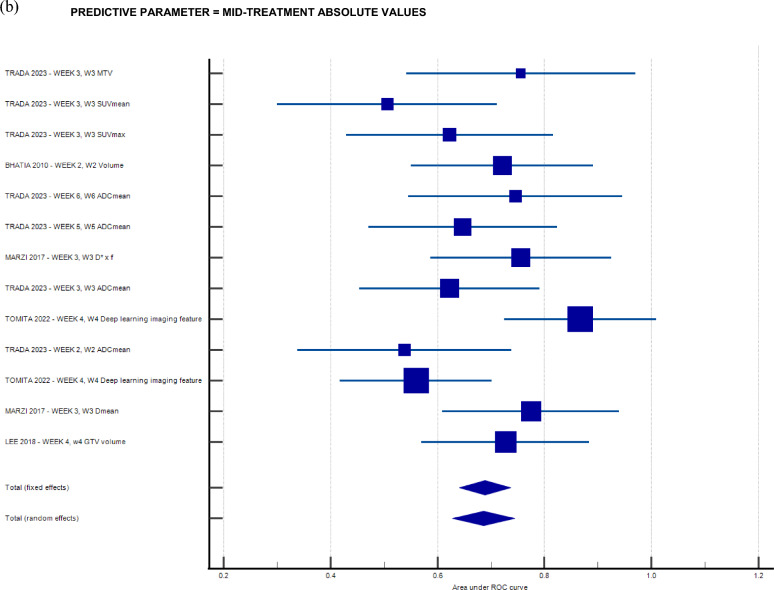

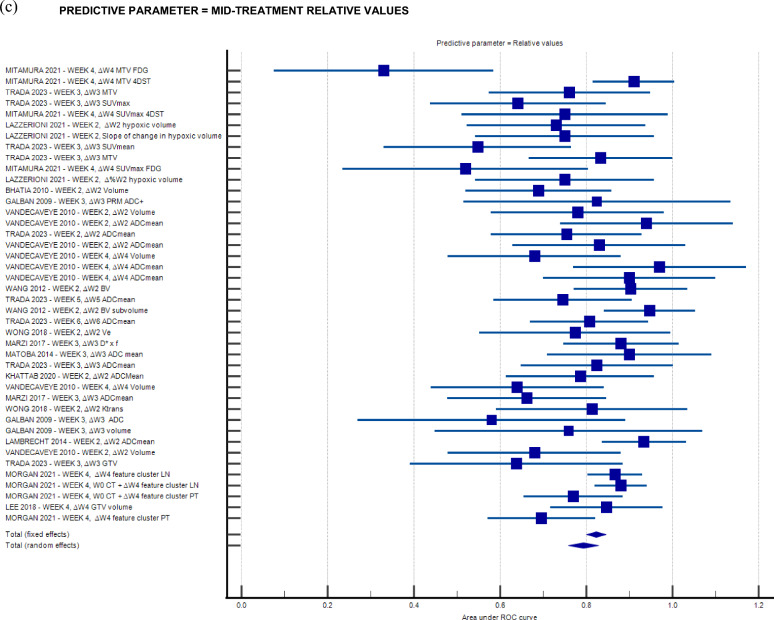
Meta-analysis. forest plots of performance of absolute baseline (**a**), absolute mid-treatment (**b**) and relative mid-treatment (**c**) imaging parameters for predicting locoregional tumour outcomes

Using a random-effects model, the pooled AUC for baseline parameters was 0.736 (95% CI: 0.688–0.785). Relative mid-treatment parameters showed improved performance (AUC 0.796; 95% CI: 0.762–0.831) compared to absolute mid-treatment parameters (AUC 0.686; 95% CI: 0.628–0.745) (Fig. 3, Supplementary Table 1).

**Fig. 4:**
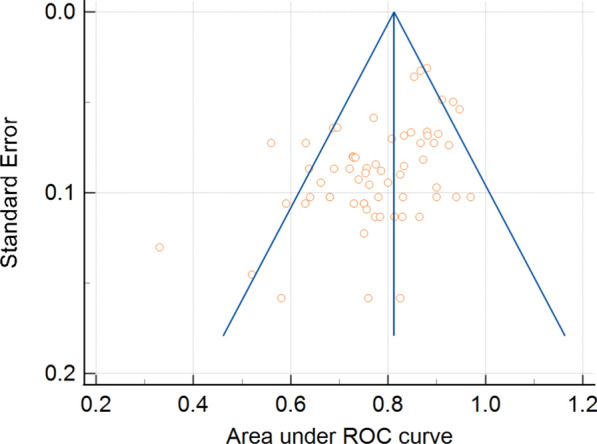
Funnel plot of meta-analysis of all quantitative imaging parameters for locoregional tumour response outcome with pseudo 95% confidence intervals. Higgins’ I^2^ index test for heterogeneity = 46.8%

A test of heterogeneity was low across all parameters included in the meta-analysis (I^2^ = 46.8%) (Fig. 4). When stratified, baseline features showed moderate heterogeneity (I^2^ = 51.5%), while absolute (I^2^ = 34.6%) and relative (I^2^ = 47.9%) mid-treatment parameters demonstrated low heterogeneity (Supplementary Fig. 1). Heterogeneity assessed using the Higgins’ I^2^ statistic (I^2^ < 25%: negligible; 25–50%: low; 50–75%: moderate; > 75%: high heterogeneity).

## Discussion

This systematic review and meta-analysis evaluated the prognostic value of quantitative mid-treatment imaging biomarkers in predicting locoregional tumour control in patients with HNSCC undergoing definitive radiotherapy. Our findings affirm the potential of mid-treatment imaging as a clinically relevant early-response biomarker. Across 41 eligible studies, mid-treatment imaging particularly relative changes (Δ) from baseline parameters were consistently associated with improved prediction of treatment outcomes. The meta-analysis of 17 studies involving 612 patients demonstrated a pooled AUC of 0.796 for relative mid-treatment parameters, compared with 0.736 for baseline and 0.686 for absolute mid-treatment values, suggesting that dynamic biological changes captured during therapy provide stronger prognostic insight than static measurements. Among imaging modalities, DWI-MRI and FDG-PET emerged as the most frequently studied and predictive. However, significant heterogeneity exists across studies in terms of imaging acquisition, timing, parameter extraction, and outcome reporting, which currently limits clinical implementation.

MRI particularly diffusion-weighted MRI (DWI), was the most studied and consistently predictive for tumour outcomes. Across multiple studies, relative change in ADC_mean_ (ΔADC_mean_) emerged as a robust imaging biomarker, with optimal cut-offs ranging from 16 to 33% and AUC values as high as 0.97. Seven DWI studies by Khattab et al. (AUC 0.786), Kim et al. AUC 0.88), Marzi et al. (AUC 0.662), Matoba et al. (AUC 0.900), Trada et al. (AUC 0.825), Vandecaveye et al. (AUC 0.97), and Wong et al. (AUC 0.937) demonstrated strong correlation with locoregional tumour control [31, 43–45, 48, 50, 53]. These consistent findings reflect the ability of ΔADC_mean_ to quantify early cytotoxic effects of radiotherapy, such as reduced tumour cellularity and increased water diffusion due to processes such as progressive necrosis, with successful treatment. A significant challenge in applying ADC-based biomarkers across centres is the variability introduced by differences in scanner hardware, acquisition protocols, and b-value selection, which can lead to inconsistent absolute ADC values.(reference) This variability limits the generalisability of absolute ADC thresholds and complicates multi-institutional standardisation. In contrast, relative mid-treatment changes in ADC (i.e., ΔADC_mean_) performed on the same scanner offer a degree of self-normalisation, as each patient effectively serves as their own internal control. This inherent normalisation reduces variability and enhances reproducibility, making relative ADC metrics more suitable for wider clinical adoption. Moreover, DWI-based histogram and textural analyses were applied in several studies (e.g., Tomita et al., Scalco et al.), suggesting an evolving role for radiomic and AI-based approaches to further refine predictive performance [36, 47].

PET imaging, particularly FDG-PET, also demonstrated strong predictive value, with relative volume based parameters ΔMTV and ΔTLG frequently emerging as the most informative features. Several studies (e.g., Lin et al., Trada et al., Wong et al.) reported ΔMTV cut-offs ranging from 33 to 75% to be predictive of locoregional control, with AUC values up to 0.833 [29, 49, 53]. Biologically, a decrease in metabolic tumour volume during treatment likely reflects a combination of tumour cell death, reduced glucose metabolism, and improved oxygenation, and may serve as an early surrogate for radiosensitivity. Conversely, a lack of significant metabolic reduction may indicate radioresistant subvolumes, which could be targeted for dose escalation. One of the key advantages of FDG-PET is its relative widespread clinical availability and established role in diagnostic and post-treatment imaging, which has led to greater standardisation of acquisition protocols across scanners compared to other functional imaging modalities. FDG-PET also offers practical advantages in clinical implementation, including the use of semi-automated region-of-interest (ROI) segmentation, which reduces inter-observer variability and enhances reproducibility. These features, combined with the potential for integration with automated workflows, make FDG-PET a feasible platform for real-time response assessment and personalised adaptive radiotherapy treatment planning in routine practice.

Emerging novel PET tracers targeting hypoxia (FMISO, FHX4, FETNIM), and proliferation (FLT, 4D-ST) add physiological specificity. For instance, Sanduleanu et al. found that residual hypoxic volume on FHX4 PET at week 2 correlated with local control (p = 0.028) [35], while Lazzeroni et al. found a ΔHTV threshold < 44.3% on FMISO PET (AUC 0.75) was predictive of recurrence [27]. Similarly, Mitamura et al. demonstrated high predictive value for ΔPTV (AUC 0.91) and ΔTLP (AUC 0.89) using 4D-ST PET [46]. While these tracers are not yet widely adopted, they provide granular insight into treatment-resistant subvolumes, offering targets for future radiotherapy dose-painting strategies.

In contrast, results from CT-based studies were more mixed. Among nine studies, six reported global volumetric changes with highly variable correlation to outcomes. Kabarriti et al. found a ΔCT volume of 19% at week 3 significantly predicted locoregional outcomes (HR 0.26) [22], while Lee et al. reported both absolute and ΔCT volumes at week 4 to be prognostic (AUCs 0.847 and 0.725, respectively) [28]. Conversely, other studies (e.g., Arens et al., Mishra et al., and Trada et al.,) found no significant correlations [32, 40, 49]. The relatively poor soft tissue contrast of CT, dependence on manual ROI delineation with limited reproducibility, and lack of standardised acquisition protocols likely contribute to the inconsistent performance of CT-based biomarkers and limit their applicability in multicentre settings. However, CT texture analysis (Morgan et al.) and perfusion CT parameters (Truong et al., Ursino et al.) showed promise, suggesting that advanced feature extraction from routine imaging may salvage prognostic utility from standard CT [34, 37, 38].

This study represents the first meta-analysis to quantitatively synthesise ROC-based AUC values for predictive performance of mid-treatment imaging in HNSCC. The use of ROC-based AUC measures offers a robust and standardised approach to assess the discriminatory ability of imaging biomarkers, independent of specific thresholds or prevalence, making it particularly suitable for comparing performance across heterogeneous studies. In our pooled meta-analysis, the area under the curve (AUC) for baseline imaging parameters was 0.736 (95% CI: 0.688–0.785). Relative mid-treatment parameters demonstrated numerically superior performance with an AUC of 0.796 (95% CI: 0.762–0.831); however, the overlapping confidence intervals indicate this difference did not reach statistical significance. This result should be interpreted with caution given that our analysis was not conducted with the aim of comparing the utility of mid-treatment imaging parameters compared to their corresponding baseline imaging parameters. In contrast, relative mid-treatment parameters demonstrated significantly higher performance with an AUC of 0.796 (95% CI: 0.762–0.831) compared to absolute mid-treatment parameters with an AUC of 0.686 (95% CI: 0.628–0.745). While prior reviews (e.g., Martens et al. and Lin et al.) provided narrative synthesis, they did not directly compare the performance of baseline, absolute, and relative parameters [10, 52]. Our findings validate the rationale for delta imaging features, where temporal changes in imaging biomarkers during radiotherapy may reflect the biological responsiveness of individual tumours. This dynamic assessment enables early differentiation between radiosensitive and radioresistant disease, providing a surrogate marker of radiotherapy efficacy that can be projected to eventual clinical outcomes following completion of treatment. Furthermore, this review highlights the increasing incorporation of multi-parametric, multi-modality models, where features from DWI, PET, and CT can be combined to improve prognostic accuracy.

Mid-treatment imaging has clear potential for personalised treatment adaptation. For responders, de-intensification strategies could mitigate long-term toxicities, while for non-responders, early escalation or surgical salvage may be indicated. Mid-treatment parameters may also assist in adaptive radiotherapy planning, identifying tumour sub-volumes for radiotherapy dose boost or omitting high-dose to regressing areas. Importantly, relative mid-treatment parameters may offer inherent self-normalisation, minimising inter-patient variability and scanner-related noise. Yet, challenges to clinical integration remain. These include a lack of standardised imaging protocols, diverse segmentation approaches, and variability in analytical pipelines. The reproducibility and interpretability of complex features, especially in multi-institution settings also limit generalisability. Thus, prioritising simple, reproducible metrics (e.g., ΔADC_mean_, ΔMTV) may be a pragmatic step toward clinical translation.

Several limitations must be acknowledged. First, individual study sample sizes were relatively small, and most lacked external validation. Only a subset (17 of 41) reported ROC-based AUC values, limiting the meta-analysis scope. Second, publication and selection biases likely inflated performance metrics. Studies that did not find significant correlations were less likely to report full statistical metrics or be published at all. Additionally, we excluded nasopharyngeal carcinoma and adjuvant radiotherapy studies due to differing biology and treatment protocols, limiting generalisability. Finally, there was substantial heterogeneity in imaging acquisition, time-points (week 1–7), and ROI methods, which restricts pooled interpretation. Future studies should pursue large, prospective multi-centre trials with harmonised imaging protocols, clearly defined mid-treatment timepoints, and publicly available datasets for external validation. Furthermore, multi-modality imaging platforms such as PET/MRI may unlock new opportunities for combining anatomical, functional, and molecular imaging to guide adaptive radiotherapy.

## Conclusions

Mid-treatment quantitative imaging offers prognostic value in predicting locoregional control in mucosal head and neck squamous cell carcinoma treated with definitive radiotherapy. This systematic review and meta-analysis demonstrate that relative mid-treatment imaging parameters outperform both baseline and absolute mid-treatment measures, with DWI-MRI (ΔADC_mean_) and FDG-PET (ΔMTV, ΔTLG) emerging as the most consistent and clinically promising biomarkers.

Despite encouraging results, substantial methodological heterogeneity and lack of standardised imaging protocols currently limit clinical translation. Future research should focus on large, prospective, multi-centre studies with harmonised imaging protocols, robust feature extraction pipelines, and external validation of predictive models. Integration of multi-parametric imaging, radiomics, and AI-based tools into clinical workflows may be required to realise the potential of mid-treatment imaging as a decision-support tool for adaptive radiotherapy in head and neck cancer.

## Supplementary Information

Additional file 1.

Additional file 2.

## Data Availability

The datasets used and/or analysed during the current study are available from the corresponding author on reasonable request.
