# Supplementary material for: Quantitative mid-treatment imaging biomarkers for response prediction after radiotherapy in head and neck cancer: a systematic review and meta-analysis
Source: EJNMMI Res. 2026 Jun 6;16:124. doi: 10.1186/s13550-026-01446-4 (PMC13457539; doi:10.1186/s13550-026-01446-4)
Supplement: Supplementary file 1 [file 13550_2026_1446_MOESM1_ESM.docx]

Supplementary table 1: Forest plot tables of performance of imaging parameters for predicting locoregional tumour outcomes

| Study, parameters: Baseline | ROC Area | Standard Error | 95% CI | z | P | Weight (%) | |
| --- | --- | --- | --- | --- | --- | --- | --- |
|  |  |  |  |  |  | Fixed | Random |
| TRADA 2023 - WEEK 0, W0 MTV | 0.589 | 0.113 | 0.368 to 0.810 |  |  | 2.19 | 3.22 |
| TRADA 2023 - WEEK 0, W0 SUVmean | 0.548 | 0.100 | 0.352 to 0.744 |  |  | 2.79 | 3.76 |
| TRADA 2023 - WEEK 0, W0 SUVmax | 0.564 | 0.104 | 0.360 to 0.768 |  |  | 2.58 | 3.58 |
| TRADA 2023 - WEEK 0, W0 MTV | 0.636 | 0.113 | 0.415 to 0.857 |  |  | 2.19 | 3.22 |
| LAZZERIONI 2021 - WEEK 0, W0 HTV | 0.630 | 0.106 | 0.422 to 0.838 |  |  | 2.49 | 3.50 |
| LAZZERIONI 2021 - WEEK 0, W0 SUVmax | 0.590 | 0.106 | 0.382 to 0.798 |  |  | 2.49 | 3.50 |
| WANG 2012 - WEEK 0, W0 GTV volume | 0.872 | 0.0816 | 0.712 to 1.000 |  |  | 4.19 | 4.73 |
| MARZI 2017 - WEEK 0, W0 ADCmean | 0.729 | 0.0806 | 0.571 to 0.887 |  |  | 4.29 | 4.80 |
| WONG 2018 - WEEK 0, ADCmean | 0.829 | 0.113 | 0.607 to 1.000 |  |  | 2.18 | 3.21 |
| WONG 2018 - WEEK 0, W0 Ve | 0.864 | 0.113 | 0.642 to 1.000 |  |  | 2.18 | 3.21 |
| KHATTAB 2020 - WEEK 0, ADCmean | 0.740 | 0.0926 | 0.559 to 0.921 |  |  | 3.25 | 4.13 |
| TOMITA 2022 - WEEK 0, W0 Deep learning imaging feature | 0.631 | 0.0725 | 0.489 to 0.773 |  |  | 5.30 | 5.31 |
| WANG 2012 - WEEK 0, W0 BV subvolume | 0.925 | 0.0737 | 0.781 to 1.000 |  |  | 5.13 | 5.23 |
| TRADA 2023 - WEEK 0, W0 ADCmean | 0.647 | 0.0860 | 0.478 to 0.816 |  |  | 3.77 | 4.48 |
| WONG 2018 - WEEK 0, W0 Ve | 0.783 | 0.113 | 0.561 to 1.000 |  |  | 2.18 | 3.21 |
| BHATIA 2010 - WEEK 0, W0 Volume | 0.638 | 0.0868 | 0.468 to 0.808 |  |  | 3.71 | 4.44 |
| TOMITA 2022 - WEEK 0, W0 Deep learning imaging feature | 0.894 | 0.0725 | 0.752 to 1.000 |  |  | 5.30 | 5.31 |
| MARZI 2017 - WEEK 0, W0 Dmean | 0.733 | 0.0804 | 0.576 to 0.890 |  |  | 4.32 | 4.81 |
| KIM 2009 - WEEK 0, W0 ADCmedian | 0.800 | 0.0944 | 0.615 to 0.985 |  |  | 3.13 | 4.03 |
| TRADA 2023 - WEEK 0, W0 GTV | 0.605 | 0.127 | 0.356 to 0.854 |  |  | 1.73 | 2.73 |
| MORGAN 2021 - WEEK 0, W0 CT feature cluster PT | 0.687 | 0.0643 | 0.561 to 0.813 |  |  | 6.75 | 5.88 |
| MORGAN 2021 - WEEK 0, W0 CT feature cluster LN | 0.854 | 0.0357 | 0.784 to 0.924 |  |  | 21.88 | 8.11 |
| LEE 2018 - WEEK 0, W0 GTV volume | 0.833 | 0.0684 | 0.699 to 0.967 |  |  | 5.97 | 5.59 |
| Total (fixed effects) | 0.759 | 0.0167 | 0.727 to 0.792 | 45.459 | <0.001 | 100.00 | 100.00 |
| Total (random effects) | 0.736 | 0.0248 | 0.688 to 0.785 | 29.736 | <0.001 | 100.00 | 100.00 |

| Study, parameters: Absolute mid-treatment | ROC Area | Standard Error | 95% CI | z | P | Weight (%) | |
| --- | --- | --- | --- | --- | --- | --- | --- |
|  |  |  |  |  |  | Fixed | Random |
| TRADA 2023 - WEEK 3, W3 MTV | 0.756 | 0.109 | 0.542 to 0.970 |  |  | 5.02 | 5.72 |
| TRADA 2023 - WEEK 3, W3 SUVmean | 0.506 | 0.105 | 0.300 to 0.712 |  |  | 5.43 | 6.07 |
| TRADA 2023 - WEEK 3, W3 SUVmax | 0.623 | 0.0990 | 0.429 to 0.817 |  |  | 6.10 | 6.62 |
| BHATIA 2010 - WEEK 2, W2 Volume | 0.721 | 0.0868 | 0.551 to 0.891 |  |  | 7.95 | 7.98 |
| TRADA 2023 - WEEK 6, W6 ADCmean | 0.746 | 0.102 | 0.546 to 0.946 |  |  | 5.75 | 6.34 |
| TRADA 2023 - WEEK 5, W5 ADCmean | 0.647 | 0.0900 | 0.471 to 0.823 |  |  | 7.39 | 7.59 |
| MARZI 2017 - WEEK 3, W3 D* x f | 0.756 | 0.0865 | 0.587 to 0.925 |  |  | 8.00 | 8.02 |
| TRADA 2023 - WEEK 3, W3 ADCmean | 0.623 | 0.0860 | 0.454 to 0.792 |  |  | 8.09 | 8.08 |
| TOMITA 2022 - WEEK 4, W4 Deep learning imaging feature | 0.867 | 0.0725 | 0.725 to 1.000 |  |  | 11.37 | 10.03 |
| TRADA 2023 - WEEK 2, W2 ADCmean | 0.538 | 0.102 | 0.338 to 0.738 |  |  | 5.75 | 6.34 |
| TOMITA 2022 - WEEK 4, W4 Deep learning imaging feature | 0.560 | 0.0725 | 0.418 to 0.702 |  |  | 11.37 | 10.03 |
| MARZI 2017 - WEEK 3, W3 Dmean | 0.775 | 0.0844 | 0.610 to 0.940 |  |  | 8.39 | 8.28 |
| LEE 2018 - WEEK 4, w4 GTV volume | 0.727 | 0.0798 | 0.571 to 0.883 |  |  | 9.38 | 8.91 |
| Total (fixed effects) | 0.689 | 0.0245 | 0.641 to 0.737 | 28.160 | <0.001 | 100.00 | 100.00 |
| Total (random effects) | 0.686 | 0.0298 | 0.628 to 0.745 | 23.055 | <0.001 | 100.00 | 100.00 |

| Study, parameters: Relative mid-treatment | ROC Area | Standard Error | 95% CI | z | P | Weight (%) | |
| --- | --- | --- | --- | --- | --- | --- | --- |
|  |  |  |  |  |  | Fixed | Random |
| MITAMURA 2021 - WEEK 4, ∆W4 MTV FDG | 0.330 | 0.130 | 0.0750 to 0.585 |  |  | 0.80 | 1.42 |
| MITAMURA 2021 - WEEK 4, ∆W4 MTV 4DST | 0.910 | 0.0485 | 0.815 to 1.000 |  |  | 5.75 | 4.05 |
| TRADA 2023 - WEEK 3, ∆W3 MTV | 0.761 | 0.0957 | 0.574 to 0.948 |  |  | 1.48 | 2.18 |
| TRADA 2023 - WEEK 3, ∆W3 SUVmax | 0.641 | 0.104 | 0.437 to 0.845 |  |  | 1.25 | 1.96 |
| MITAMURA 2021 - WEEK 4, ∆W4 SUVmax 4DST | 0.750 | 0.122 | 0.510 to 0.990 |  |  | 0.90 | 1.56 |
| LAZZERIONI 2021 - WEEK 2, ∆W2 hypoxic volume | 0.730 | 0.106 | 0.522 to 0.938 |  |  | 1.20 | 1.91 |
| LAZZERIONI 2021 - WEEK 2, Change in hypoxic volume | 0.750 | 0.106 | 0.542 to 0.958 |  |  | 1.20 | 1.91 |
| TRADA 2023 - WEEK 3, ∆W3 SUVmean | 0.548 | 0.111 | 0.330 to 0.766 |  |  | 1.10 | 1.79 |
| TRADA 2023 - WEEK 3, ∆W3 MTV | 0.833 | 0.0852 | 0.666 to 1.000 |  |  | 1.86 | 2.50 |
| MITAMURA 2021 - WEEK 4, ∆W4 SUVmax FDG | 0.520 | 0.145 | 0.235 to 0.805 |  |  | 0.64 | 1.20 |
| LAZZERIONI 2021 - WEEK 2, ∆%W2 hypoxic volume | 0.750 | 0.106 | 0.542 to 0.958 |  |  | 1.20 | 1.91 |
| BHATIA 2010 - WEEK 2, ∆W2 Volume | 0.689 | 0.0868 | 0.519 to 0.859 |  |  | 1.80 | 2.45 |
| GALBAN 2009 - WEEK 3, ∆W3 PRM ADC+ | 0.825 | 0.158 | 0.515 to 1.000 |  |  | 0.54 | 1.04 |
| VANDECAVEYE 2010 - WEEK 2, ∆W2 Volume | 0.780 | 0.102 | 0.580 to 0.980 |  |  | 1.29 | 2.00 |
| VANDECAVEYE 2010 - WEEK 2, ∆W2 ADCmean | 0.940 | 0.102 | 0.740 to 1.000 |  |  | 1.29 | 2.00 |
| TRADA 2023 - WEEK 2, ∆W2 ADCmean | 0.754 | 0.0890 | 0.579 to 0.929 |  |  | 1.70 | 2.37 |
| VANDECAVEYE 2010 - WEEK 2, ∆W2 ADCmean | 0.830 | 0.102 | 0.630 to 1.000 |  |  | 1.29 | 2.00 |
| VANDECAVEYE 2010 - WEEK 4, ∆W4 Volume | 0.680 | 0.102 | 0.480 to 0.880 |  |  | 1.29 | 2.00 |
| VANDECAVEYE 2010 - WEEK 4, ∆W4 ADCmean | 0.970 | 0.102 | 0.770 to 1.000 |  |  | 1.29 | 2.00 |
| VANDECAVEYE 2010 - WEEK 4, ∆W4 ADCmean | 0.900 | 0.102 | 0.700 to 1.000 |  |  | 1.29 | 2.00 |
| WANG 2012 - WEEK 2, ∆W2 BV | 0.903 | 0.0676 | 0.771 to 1.000 |  |  | 2.96 | 3.16 |
| TRADA 2023 - WEEK 5, ∆W5 ADCmean | 0.746 | 0.0820 | 0.585 to 0.907 |  |  | 2.01 | 2.61 |
| WANG 2012 - WEEK 2, ∆W2 BV subvolume | 0.947 | 0.0538 | 0.842 to 1.000 |  |  | 4.66 | 3.79 |
| TRADA 2023 - WEEK 6, ∆W6 ADCmean | 0.807 | 0.0702 | 0.669 to 0.945 |  |  | 2.75 | 3.06 |
| WONG 2018 - WEEK 2, ∆W2 Ve | 0.774 | 0.113 | 0.552 to 0.996 |  |  | 1.05 | 1.74 |
| MARZI 2017 - WEEK 3, ∆W3 D* x f | 0.881 | 0.0684 | 0.747 to 1.000 |  |  | 2.89 | 3.13 |
| MATOBA 2014 - WEEK 3, ∆W3 ADC mean | 0.900 | 0.0971 | 0.710 to 1.000 |  |  | 1.43 | 2.14 |
| TRADA 2023 - WEEK 3, ∆W3 ADCmean | 0.825 | 0.0901 | 0.648 to 1.000 |  |  | 1.67 | 2.34 |
| KHATTAB 2020 - WEEK 2, ∆W2 ADCMean | 0.786 | 0.0878 | 0.614 to 0.958 |  |  | 1.75 | 2.42 |
| VANDECAVEYE 2010 - WEEK 4, ∆W4 Volume | 0.640 | 0.102 | 0.440 to 0.840 |  |  | 1.29 | 2.00 |
| MARZI 2017 - WEEK 3, ∆W3 ADCmean | 0.662 | 0.0944 | 0.477 to 0.847 |  |  | 1.52 | 2.21 |
| WONG 2018 - WEEK 2, ∆W2 Ktrans | 0.813 | 0.113 | 0.591 to 1.000 |  |  | 1.05 | 1.74 |
| GALBAN 2009 - WEEK 3, ∆W3 ADC | 0.580 | 0.158 | 0.270 to 0.890 |  |  | 0.54 | 1.04 |
| GALBAN 2009 - WEEK 3, ∆W3 volume | 0.759 | 0.158 | 0.449 to 1.000 |  |  | 0.54 | 1.04 |
| LAMBRECHT 2014 - WEEK 2, ∆W2 ADCmean | 0.934 | 0.0497 | 0.836 to 1.000 |  |  | 5.46 | 3.99 |
| VANDECAVEYE 2010 - WEEK 2, ∆W2 Volume | 0.680 | 0.102 | 0.480 to 0.880 |  |  | 1.29 | 2.00 |
| TRADA 2023 - WEEK 3, ∆W3 GTV | 0.638 | 0.126 | 0.391 to 0.885 |  |  | 0.85 | 1.49 |
| MORGAN 2021 - WEEK 4, ∆W4 feature cluster LN | 0.867 | 0.0324 | 0.803 to 0.931 |  |  | 12.88 | 4.86 |
| MORGAN 2021 - WEEK 4, W0 CT +∆W4 feature cluster LN | 0.880 | 0.0311 | 0.819 to 0.941 |  |  | 13.95 | 4.92 |
| MORGAN 2021 - WEEK 4, W0 CT +∆W4 feature cluster PT | 0.770 | 0.0587 | 0.655 to 0.885 |  |  | 3.93 | 3.56 |
| LEE 2018 - WEEK 4, ∆W4 GTV volume | 0.847 | 0.0666 | 0.716 to 0.978 |  |  | 3.05 | 3.21 |
| MORGAN 2021 - WEEK 4, ∆W4 feature cluster PT | 0.696 | 0.0640 | 0.570 to 0.822 |  |  | 3.30 | 3.32 |
| Total (fixed effects) | 0.823 | 0.0116 | 0.800 to 0.846 | 70.800 | <0.001 | 100.00 | 100.00 |
| Total (random effects) | 0.793 | 0.0179 | 0.758 to 0.828 | 44.370 | <0.001 | 100.00 | 100.00 |

Supplementary figure 1: Funnel plot of meta-analysis of quantitative imagining parameters for locoregional tumour response outcomes with pseudo 95% confidence intervals. Baseline value (I^2^ = 51.5%); Absolute mid-treatment (I^2^ = 34.6%); Relative mid-treatment (I^2^ = 47.9%).
