# Supplementary material for: Quantitative mid-treatment imaging biomarkers for response prediction after radiotherapy in head and neck cancer: a systematic review and meta-analysis
Source: EJNMMI Res. 2026 Jun 6;16:124. doi: 10.1186/s13550-026-01446-4 (PMC13457539; doi:10.1186/s13550-026-01446-4)
Supplement: Supplementary file 2 [file 13550_2026_1446_MOESM2_ESM.docx]

**Supplementary A: Search Strategy**

PICOS strategy

P - Head and neck cancer patients

I - Radiotherapy

C - Not applicable

O - Locoregional disease control

S - Prediction studies

| **Searching keywords** | |
| --- | --- |
| # | SCOPUS  “head and neck” OR “head-and-neck” OR “oropharynx” OR “oropharyngeal” OR “pharyngeal neoplasm” OR “hypopharynx” OR “hypopharyngeal” OR “larynx” OR “laryngeal” OR “HNSCC” OR “tonsil” OR “Tonsillar” OR "Mouth Neoplasms" OR “tongue” OR “oral cavity”  PUBMED  "head and neck"[Title/Abstract] OR "head-and-neck"[Title/Abstract] OR "oropharynx"[Title/Abstract] OR "oropharyngeal"[Title/Abstract] OR "pharyngeal neoplasm"[Title/Abstract] OR "hypopharynx"[Title/Abstract] OR "hypopharyngeal"[Title/Abstract] OR "larynx"[Title/Abstract] OR "laryngeal"[Title/Abstract] OR "HNSCC"[Title/Abstract] OR "tonsil"[Title/Abstract] OR "Tonsillar"[Title/Abstract] OR "mouth neoplasms"[Title/Abstract] OR "tongue"[Title/Abstract] OR "oral cavity"[Title/Abstract] OR "head and neck neoplasms"[MeSH Terms]  EMBASE/CCTR  "head and neck".ti,ab. or "head-and-neck".ti,ab. or "oropharynx".ti,ab. or "oropharyngeal".ti,ab. or "pharyngeal neoplasm".ti,ab. or "hypopharynx".ti,ab. or "hypopharyngeal".ti,ab. or "larynx".ti,ab. or "laryngeal".ti,ab. or "hnscc".ti,ab. or "tonsil".ti,ab. or "tonsillar".ti,ab. or "mouth neoplasms".ti,ab. or "tongue".ti,ab. or "oral cavity".ti,ab. or "head and neck tumor"/ |
| #2 | SCOPUS  “radiotherapy” OR “irradiation” OR “radiation” OR “IMRT” OR “VMAT” OR “chemoradiotherapy”  PUBMED  "radiotherapy"[Title/Abstract] OR "irradiation"[Title/Abstract] OR "radiation"[Title/Abstract] OR "IMRT"[Title/Abstract] OR "VMAT"[Title/Abstract] OR "chemoradiotherapy"[Title/Abstract] OR "radiotherapy"[MeSH Terms]  EMBASE/CCTR  "radiotherapy".ti,ab. or "irradiation".ti,ab. or "radiation".ti,ab. or "imrt".ti,ab. or "vmat".ti,ab. or "chemoradiotherapy".ti,ab. or "radiotherapy"/ |
| #3 | SCOPUS  "outcome" OR "recurrence" OR "treatment outcome" OR "locoregional" OR "disease-free survival" OR "progression-free survival" OR "local failure free survival" OR “regional failure free survival” OR “loco-regional failure free survival” OR "overall survival" OR "DFS" OR "LRC" OR "response"  PUBMED  "outcome"[Title/Abstract] OR "recurrence"[Title/Abstract] OR "locoregional"[Title/Abstract] OR "disease free survival"[Title/Abstract] OR "progression free survival"[Title/Abstract] OR "local failure free survival"[Title/Abstract] OR "regional failure free survival"[Title/Abstract] OR "loco regional failure free survival"[Title/Abstract] OR "overall survival"[Title/Abstract] OR "DFS"[Title/Abstract] OR "LRC"[Title/Abstract] OR "response"[Title/Abstract] OR "treatment outcome"[MeSH Terms]  EMBASE/CCTR  "outcome".ti,ab. or "recurrence".ti,ab. or "locoregional".ti,ab. or "disease free survival".ti,ab. or "progression free survival".ti,ab. or "local failure free survival".ti,ab. or "regional failure free survival".ti,ab. or "loco regional failure free survival".ti,ab. or "overall survival".ti,ab. or "dfs".ti,ab. or "lrc".ti,ab. or "response".ti,ab. or "treatment outcome"/ |
| #4 | SCOPUS  “mid-treatment” OR “mid treatment” OR “during therapy” OR “serial” OR “intra-treatment” OR “intra treatment” OR “intratreatment” OR “intratherapy” OR “intra-therapy” OR “intra therapeutic” OR “intra-therapeutic” OR “early response” OR “change” OR “interim”  PUBMED  "mid-treatment"[Title/Abstract] OR "mid-treatment"[Title/Abstract] OR "during therapy"[Title/Abstract] OR "serial"[Title/Abstract] OR "intra-treatment"[Title/Abstract] OR "intra-treatment"[Title/Abstract] OR "intratreatment"[Title/Abstract] OR "intratherapy"[Title/Abstract] OR "intra-therapy"[Title/Abstract] OR "intra-therapeutic"[Title/Abstract] OR "intra-therapeutic"[Title/Abstract] OR "early response"[Title/Abstract] OR "change"[Title/Abstract] OR "interim"[Title/Abstract]  EMBASE/CCTR  "mid-treatment".ti,ab. or "mid-treatment".ti,ab. or "during therapy".ti,ab. or "serial".ti,ab. or "intra-treatment".ti,ab. or "intra-treatment".ti,ab. or "intratreatment".ti,ab. or "intratherapy".ti,ab. or "intra-therapy".ti,ab. or "intra-therapeutic".ti,ab. or "intra-therapeutic".ti,ab. or "early response".ti,ab. or "change".ti,ab. or "interim".ti,ab. |
| #5 | SCOPUS  "FDG" OR "positron emission tomography" OR "Fluorodeoxyglucose" OR "Fludeoxyglucose" OR "deoxyglucose" OR "desoxyglucose" OR "deoxy-glucose" OR "desoxy-glucose" OR "deoxy-d-glucose" OR "desoxy-d-glucose" OR "2deoxyglucose" OR "fluorodeoxyglucose" OR "fluorodesoxyglucose" OR "fludeoxyglucose" OR "fluordeoxyglucose" OR "fluordesoxyglucose" OR "FMISO" OR "FAZA" OR "Fluoromisonidazole" OR "CT" OR "tomography" OR "dynamic contrast" OR "MRI" OR "magnetic resonance imaging" OR "diffusion weighted" OR "DWI" OR "DCE" OR "multiparametric" OR "MR-Diffusion" OR "fMRI" OR "NMR" OR "nuclear magnetic" OR "Image Enhancement/methods" OR "Magnetic Resonance Spectroscopy" OR "MRS"  PUBMED  "FDG"[Title/Abstract] OR "positron emission tomography"[Title/Abstract] OR "Fluorodeoxyglucose"[Title/Abstract] OR "Fludeoxyglucose"[Title/Abstract] OR "deoxyglucose"[Title/Abstract] OR "desoxyglucose"[Title/Abstract] OR "deoxy-glucose"[Title/Abstract] OR "desoxy-glucose"[Title/Abstract] OR "deoxy-d-glucose"[Title/Abstract] OR "desoxy-d-glucose"[Title/Abstract] OR "2deoxyglucose"[Title/Abstract] OR "Fluorodeoxyglucose"[Title/Abstract] OR "fluorodesoxyglucose"[Title/Abstract] OR "Fludeoxyglucose"[Title/Abstract] OR "fluordeoxyglucose"[Title/Abstract] OR "fluordesoxyglucose"[Title/Abstract] OR "FMISO"[Title/Abstract] OR "FAZA"[Title/Abstract] OR "Fluoromisonidazole"[Title/Abstract] OR "CT"[Title/Abstract] OR "tomography"[Title/Abstract] OR "dynamic contrast"[Title/Abstract] OR "MRI"[Title/Abstract] OR "magnetic resonance imaging"[Title/Abstract] OR "diffusion weighted"[Title/Abstract] OR "DWI"[Title/Abstract] OR "DCE"[Title/Abstract] OR "multiparametric"[Title/Abstract] OR "MR-Diffusion"[Title/Abstract] OR "fMRI"[Title/Abstract] OR "NMR"[Title/Abstract] OR "nuclear magnetic"[Title/Abstract] OR "image enhancement methods"[Title/Abstract] OR "magnetic resonance spectroscopy"[Title/Abstract] OR "MRS"[Title/Abstract] OR "diagnostic imaging"[MeSH Terms]  EMBASE/CCTR  "fdg".ti,ab. or "positron emission tomography".ti,ab. or "fluorodeoxyglucose".ti,ab. or "fludeoxyglucose".ti,ab. or "deoxyglucose".ti,ab. or "desoxyglucose".ti,ab. or "deoxy-glucose".ti,ab. or "desoxy-glucose".ti,ab. or "deoxy-d-glucose".ti,ab. or "desoxy-d-glucose".ti,ab. or "2deoxyglucose".ti,ab. or "fluorodeoxyglucose".ti,ab. or "fluorodesoxyglucose".ti,ab. or "fludeoxyglucose".ti,ab. or "fluordeoxyglucose".ti,ab. or "fluordesoxyglucose".ti,ab. or "fmiso".ti,ab. or "faza".ti,ab. or "fluoromisonidazole".ti,ab. or "ct".ti,ab. or "tomography".ti,ab. or "dynamic contrast".ti,ab. or "mri".ti,ab. or "magnetic resonance imaging".ti,ab. or "diffusion weighted".ti,ab. or "dwi".ti,ab. or "dce".ti,ab. or "multiparametric".ti,ab. or "mr-diffusion".ti,ab. or "fmri".ti,ab. or "nmr".ti,ab. or "nuclear magnetic".ti,ab. or "image enhancement methods".ti,ab. or "magnetic resonance spectroscopy".ti,ab. or "mrs".ti,ab. or "diagnostic imaging"/ |

Filters

- Published after 2005 (3D PET, lack of quantitative analysis)
- Human studies
- English

Search results: Sep 23

| **Database** | **Query** (Search date: 28 Sep 2023) | **Results** |
| --- | --- | --- |
| PubMed | #1 AND #2 AND #3 AND #4 AND #5 | 402 |
| Embase | #1 AND #2 AND #3 AND #4 AND #5 | 577 |
| Scopus | #1 AND #2 AND #3 AND #4 AND #5 | 947 |
| CCTR | #1 AND #2 AND #3 AND #4 AND #5 | 46 |
